# I Can Stand Learning: A Controlled Pilot Intervention Study on the Effects of Increased Standing Time on Cognitive Function in Primary School Children

**DOI:** 10.3390/ijerph15020356

**Published:** 2018-02-17

**Authors:** Katharina Wick, Oliver Faude, Susanne Manes, Lukas Zahner, Lars Donath

**Affiliations:** 1 Institute of Psychosocial Medicine and Psychotherapy, University Hospital, Friedrich-Schiller-University Jena, 07743 Jena, Germany; susanne.manes@med.uni-jena.de; 2 Department of Sport, Exercise and Health, University of Basel, 4052 Basel, Switzerland; oliver.faude@unibas.ch (O.F.); lukas.zahner@unibas.ch (L.Z.); 3 Institute of Exercise Training and Computer Science in Sport, German Sport University Cologne, 50933 Köln, Germany; l.donath@dshs-koeln.de

**Keywords:** physical activity, sit-stand desk, sedentarism, self-reported data, subjective measures, objective measures, bias, cognitive function, school setting, adolescence

## Abstract

Sedentarism is considered an independent cardiovascular risk factor. Thus, the present study investigated the effects of employing standing desks in classrooms on cognitive function. The intervention class (IG; *n* = 19) was supplied with standing desks and balance pads for 11 weeks. The control class (CG; *n* = 19) received lessons as usual. Standing time was assessed objectively (accelerometers) and subjectively (self-report sheets, external classroom observers). The impact of standing on the digit span task and Eriksen flanker task was analysed. The standing time of the IG was higher during the school day in comparison to the CG (lesson: *p* = 0.004; break: *p* = 0.003). The intra-class correlation coefficient between self-reports and external observation was high (ICC = 0.94). The IG improved slightly on the Digit Span Task compared to CG. Employing standing desks for at least 1 h per school day serves as a feasible and effective opportunity to improve cognitive function.

## 1. Introduction

Ranked as the fourth leading risk factor for global mortality, responsible for 6% of the deaths worldwide and contributing to nearly 60% of non-communicable diseases [1], physical inactivity (PI) has been entitled the “biggest public health problem of the current century” [2]. Global physical activity (PA) recommendations for children suggest 60 min of moderate-to-vigorous daily PA [3]. Furthermore, international PA guidelines for young children (e.g., Canada, UK and Australia) recommend 3 h of at least light PA a day [4]. It has been shown that only every second child seems to meet these recommendations [5]. According to the European Youth Heart study, 80% of children are not meeting the minimum recommended PA levels in Europe [6]. Sixty minutes of at least moderate PA are recommended in order to reduce cardiovascular risk [7].

Sedentary behavior is considered an independent risk factor for the development of various disease conditions and notably contributes to daily PI patterns [8,9]. Beside PI during leisure time, occupational sitting is linked to higher risk of morbidity and all-cause mortality [10]. In children, adverse associations between sedentary behavior (sitting) and cardio-metabolic health risk markers (obesity, blood pressure, cholesterol, insulin), fitness, cognitive development and academic achievement have been reported [11,12]. The school setting characteristically comprises high volumes of sitting: About two-thirds of the class time is spent sitting [13]. Children sit longer during school time compared to non-school time on school days [14]. In this regard, Hinckson et al. [15] found sitting time reductions of ∼1 h/day in pupils using sit-to-stand desks. Comparable reductions around 10% (50 min sitting time reductions) were observed by a pilot study conducted in the UK [16]. Several classroom interventions in children aiming at reducing sitting time showed notable reductions of sitting time and increases in energy expenditure [15,17,18,19,20].

The impact of standing desks on cognitive performance and academic achievement has been recently summarized [21,22]. These studies revealed inconsistent results depending on age-groups: For example, Torbeyns et al. [21] did not find relevant effects on work performance, cognitive function and computer task performance in adults. In contrast Mura et al. [22] observed positive results on academic achievement and cognitive skills in school-aged children.

Against this background, the present pilot study investigated the applicability of standing desks in classrooms targeting 1 h of daily standing time. Additionally, we hypothesized that 1 h of standing time results in meaningful improvements of cognitive performance within 11 intervention weeks. We therefore differentiated between standing and walking time during lessons and breaks and assessed working memory and inhibition. From a perspective of practicability, we also examined whether children do record their standing times correctly compared to an external adult classroom observer.

## 2. Materials and Methods

### 2.1. Participants

Healthy Swiss children aged between 10 to 12 years took part in this non-randomized, controlled, interventional pilot study. The study involved a total of 38 children in two classes. One class served as the intervention group (IG; *n* = 19), the other as the control group (CG; *n* = 19). Classes were recruited from two different primary schools, both from the rural area near Basel with a comparable socioeconomic background.

The intervention lasted for 11 weeks, from August to December 2014, without the vacation period. The teachers were asked to encourage pupils to work for about 60 min a day at the standing desks. Pupils stood on an unstable surface while standing at the desk. The teacher reminded the children regularly to use the standing desks during the lessons, but the children were allowed to manage their standing time by themselves. Once a child was standing, the standing time needed to be maintained for at least 10 min. The control class attended their regular classes (lessons as usual) in those 11 weeks with no restrictions regarding the activity level of the lessons.

Prior to the start of the study, we informed the participants and their parents about the study process and the voluntariness of participation. We obtained the signed, informed, written consent of the parents and their children. The study protocol was approved by the local ethics committee (Ethikkommission Zentral- und Nordwestschweiz, approval number 2014/162) and complied with the declaration of Helsinki.

The demographic characteristics of the participants are presented in Table 1.

To evaluate the accuracy of self-reported standing time in comparison to external observation in the IG only, three students were excluded from this part of the study. Two students were excluded due to very limited presence in the classroom, due to their attending special education courses in other rooms. A third student showed large uncertainty in the use of the self-report sheet due to low German language skills.

### 2.2. Instruments and Procedure

#### 2.2.1. Standing Desk and Floor Mat

**Figure 1:**
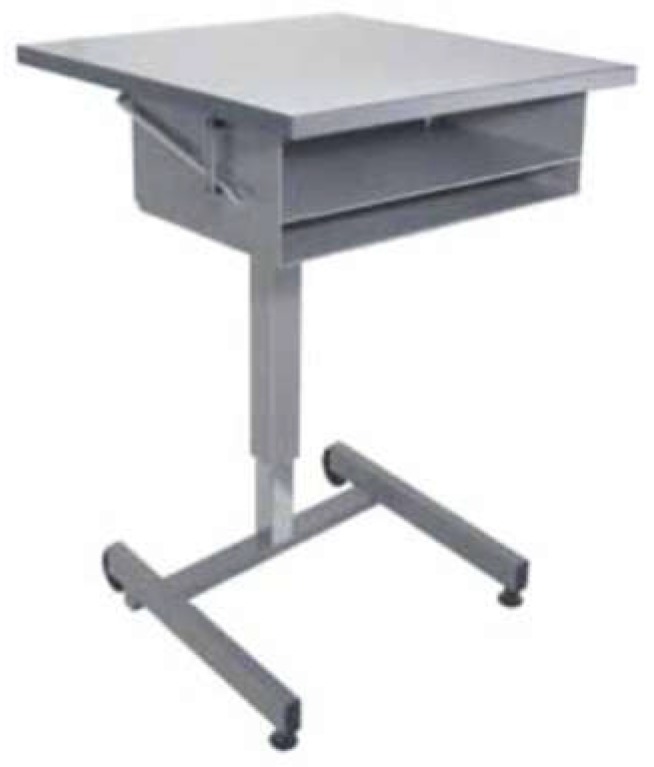
Model of the used standing desk adopted by Hüba AG.

The standing desk Hüba-Ergofit 03.AF.OK.FMH.R of the Hüba AG in Lucerne, Switzerland, was used (see Figure 1). The desk top had the dimensions 650 × 650 mm and they could be easily fixed at a height between 750 and 1200 mm. In addition to the standard model, a support tool for the floor mat was attached to the back of each desk.

While standing at the desk, the subjects stood on an unstable surface. A floor mat of Müller Schaumstoff GmbH in Liestal, Switzerland, (dimensions 450 × 450 × 60 mm) fit between the two desk legs. To avoid accidents, a non-slippery layer was applied underneath the mat.

#### 2.2.2. Objective Measurement of Activity

The body position (sitting, standing, walking) of the participants was objectively measured using the ActiGraph wGT3X-BT (ActiGraph LLC., Pensacola, FL, USA) during five schooldays.

This device showed high sensitivity (~99%) and specificity (100%) in distinguishing between sitting, standing, walking, running, and cycling when worn at the thigh, described in detail elsewhere [23]. The small triaxial accelerometer has an integrated inclinometer function using raw data classifying activities as sitting, standing, and moving [24]. The devices were initialized to record data at a sampling rate of 60 Hz for all three axes. Using ActiLife software (Version 6.10.1, ActiGraph LLC., Pensacola, FL, USA)) all data could be downloaded and a classification of wear time could be determined according to Choi et al. [25] For data processing an epoch length of 60 s was used. All participants received a detailed instruction in the use of the devices by the study team. The ActiGraph wGT3X-BT (ActiGraph LLC., Pensacola, FL, USA) was attached with an elastic belt at the right thigh. Each accelerometer was pre-initialized with technical and personal data. The period of data recording began on Monday morning at 7.00 a.m. and ended on Friday afternoon at 5.00 p.m. The accelerometer was carried by the children during the week throughout the school day. It was permitted to remove the ActiGraph during physical education, lunch break and after school. The study team or the teacher checked whether the accelerometer was attached to the body in the correct position. The body position was provided in minutes and percentages of the total time spent in school. The standing time was analyzed separately for lessons (including short breaks of 5 min duration) and big breaks (about 15 min) in the morning and afternoon.

#### 2.2.3. Subjective Measurement of Activity: Self-Reporting

In order to record the standing time of the children by self-reporting, each child received a self-report sheet. At the beginning of every week, the self-report sheets were placed by study assistants or the teacher on the desks of all children. Each timeline was divided into units of 10 min. It was possible to plot a line in the middle of two lines, precisely to 5 min. To facilitate the recordings, the lunch breaks were entered in the timeline. Further, only those times were listed in which the students had the opportunity to work at the standing desks, comprising 775 min during one school week. At the beginning of the first intervention week, study assistants explained the usage of the self-report sheets as follows: When changing from the sitting position to standing, they had to enter an arrow pointing upwards (↑). Conversely, they had to add the change from the standing position to sitting with a down arrow (↓). The first week was accompanied by at least one investigator to answer further questions.

##### Subjective Measurement of Activity: External Observation

For four weeks of the 11 intervention weeks, study assistants recorded the standing time of the pupils in the IG. During these four weeks, the students wore an accelerometer for five days. The CG was only accompanied by study assistants during the accelerometer measurement.

#### 2.2.4. Digit Span Task

With the digit span task, both the working memory (backward digit span) as well as short-term memory (forward digit span) can be tested [26]. Participants first had to repeat a series of numbers in order, followed by repeating a series of numbers in reverse order (“WAIS-like (default)” configuration, two trials each, WAIS = Wechsler Adult Intelligence Scale). The length of the sequence number was constantly increased by one number in the course of the experiment, if the subjects answered right at the first and/or the second trial. Only two wrong answers were allowed. The time window in which the subjects had to answer was not limited. The maximum attained sequence length on digit span backward and the sum of correct trials on digit span forward was analyzed.

#### 2.2.5. Eriksen Flanker Task

Inhibitory control was assessed with the Eriksen flanker task (Eriksen flanker task (default) configuration) which measures the ability to suppress distractors and attend relevant information [27]. Subjects were asked to respond as quickly and accurately as possible with a key press depending on the direction of a centrally presented arrow (left or right), which was showed always in the same place above a fixation point. The arrow was flanked by congruous (facing in the same direction) or incongruous (facing the opposite direction) arrows, which had to be ignored. After 10 test trials, participants completed three series of 100 experimental trials with equiprobable and random congruency and incongruency (each 150 trials). The fixation point was shown for 1000 ms and the arrows were presented for 200 ms with interstimulus intervals of 1000, 1250, 1500, 1750 and 2000 ms. Answers had to be given within 1000 ms. Measures of median reaction time (in ms) and accuracy (in%, range 0–1) were analyzed.

#### 2.2.6. Body Composition

In order to determine the weight of the participants the InBody 170 (JP Global Markets GmbH, Eschborn, Germany) was used. Due to practical benefits and privacy reasons, all children were weighted with their normal clothes and went barefoot on the contact surfaces. To measure the size a measuring pole was used. The children stood upright, facing forward, with the shoulders and heels on the wall.

### 2.3. Data Analysis

All calculations were performed with SPSS (version 22.0; SPSS Inc., Chicago, IL, USA). In order to compare the objectively measured sitting, standing, and walking time during lessons and breaks between IG and CG independent t-tests were computed. Additionally, the effect size d was calculated according to Cohen. The observed effects were classified according to Cohen [28] into small (d > 0.2), moderate (d > 0.5), and large (d > 0.8).

To describe the relationship between the subjectively measured standing time (self vs. external report), a paired *t*-test was computed to examine the difference between the measures. Furthermore, an intra-class correlation coefficient (ICC) was calculated. The ICC value was rated as poor (0 to 0.39), moderate (0.4 to 0.74), or excellent (0.75 to 1) [29].

A Bland–Altman-plot was created to estimate heteroscedasticity and to describe the deviation between the subjective measures (self vs. external report). The plot shows the systematic bias (mean difference between subjective and objective measures) and the limits of agreement (±1.96* standard deviation of the difference between both measures) to obtain a 95% random error component.

Due to the longitudinal design of the measures in the digit span task and Eriksen flanker task a 2 (control vs. intervention group) × 2 (pre vs. post measurement) analysis of variance (ANOVA) was conducted. To estimate the effect size the partial Eta^2^ was calculated. The observed effects could be classified into small (η*p*^2^ > 0.01), moderate (η*p*^2^ > 0.06), and large (η*p*^2^ > 0.14).

The significance level for all tests was set at 0.05 a priori.

## 3. Results

### 3.1. Objectively Measured Sitting, Standing, and Walking Time during Lessons and Breaks

Objectively measured by accelerometer, all participants sat on average 179 (±18) min, stood 54 (±15) min and walked 19 (±5) min of their teaching time on an average day. The measured sitting and standing time of the IG during lessons differed in comparison to the CG. During breaks, all pupils sat 7.0 (±2.1) min, stood 7.6 (±2.7) min and walked 6.3 (±2.7) min per day. The measured standing time of the IG during breaks differed in comparison to the CG due to 5 min longer breaks. The percentage distribution of standing times in breaks in the two groups is comparable. Table 2 summarizes the comparison of objectively measured sitting, standing, and walking time between IG and CG, separately for lessons and breaks.

### 3.2. Association between Self-Reported Desk Standing Time

**Figure 2:**
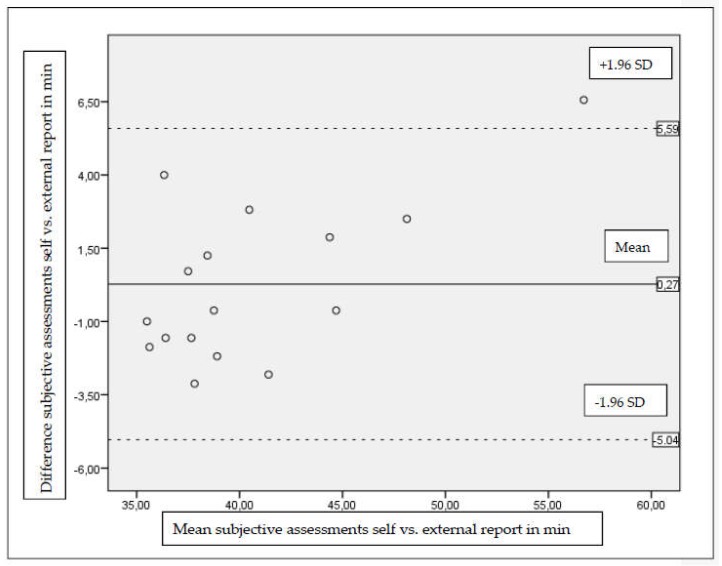
Comparison between the two subjective measures (self vs. external report) of standing time using Bland-Altman plots. The chart presents the mean difference (intermediate line) and ±1.96* standard deviation of difference (upper and lower line), showing the limits of agreement.

Participants noted on average 40.7 (±6.5) min of standing time per day on their control sheet (self-report). The external observers recorded 40.4 (±4.9) min. The comparison of the two subjective assessments yielded similar results of self-report and external report (*t*(15) = −0.40, *p* = 0.70). The intra-class correlation coefficients between both subjective measure were excellent (ICC = 0.94). The Bland-Altman analysis is shown in Figure 2: Comparison between the two subjective measures (self vs. external report) of standing time using Bland-Altman plots. The chart presents the mean difference (intermediate line) and ±1.96* standard deviation of difference (upper and lower line), showing the limits of agreement.

We were not able to compare subjective (self-report and external report) and objective data (accelerometer) due to different measurement periods, which were not documented by the research assistants.

### 3.3. Digit Span Task

For the digit span task, the number of correct trials, both groups improved moderately over time (forward: F(1; 36) = 3.41, *p* = 0.07, ηp^2^ = 0.09; backward: F(1; 36) = 3.50, *p* = 0.07, ηp^2^ = 0.09). No group influence (IG vs. CG) could be observed from the first measurement point to the second measurement point. Based on the pre-measurement, descriptive improvements could be found at post measurement for the IG compared to the CG. The mean changes and their effect sizes were higher in the IG (IG: 0.46–0.48; CG: 0.26) for the digit span task. The group × time interaction was neither significant for the short-term memory (forward digit span) nor for the working memory (backward digit span). For Eriksen flanker task, the changes in reaction time were influenced by time (pre vs. post), showing that both groups did (marginally) improve over time (congruous: F(1; 34) = 4.41, *p* = 0.04, ηp^2^ = 0.12; incongruous: F(1; 34) = 3.39, *p* = 0.07, ηp^2^ = 0.09). These improvements over time could also be found for accuracy (congruous: F(1; 34) = 8.20, *p* = 0.01, ηp^2^ = 0.19; incongruous: F(1; 34) = 3.86, *p* = 0.06, ηp^2^ = 0.10). For the group and group × time interactions no meaningful results could be found. Table 3 lists each mean value of all outcomes concerning the digit span task and Eriksen flanker task.

## 4. Discussion

The present study aimed at examining the establishment of 60 min usage of standing desks in classrooms and their impact on cognitive function within 11 weeks in primary school children.

We observed that almost three fourths of the time in class was spent sitting, less than 20% standing, and 7.5% walking in the CG. This equals more than 3 h of sitting, 45 min of standing, and almost 20 min walking within a school day. Similar volumes of sitting time (at least two-thirds of class time) have been reported in other studies [30,31].

Compared to the CG, the IG sat 13 min less per day (65 min per week). Effect sizes ranged between 0.75 and 1.04. This resembles results of other studies applying standing desks to pupils, summarized in a review by Minges et al. [32] They found notable standing time increases (effect sizes: 0.38–0.71), while weekly sitting time decreased by approximately 60 min (effect sizes: 0.27–0.49).

The IG had the opportunity to use the standing desks for 775 min during one week. The accelerometer data reported a standing time of 302′5″ min at the desks (60.5 daily min). This corresponds to 39% of usage. In comparison, prompt-studies in adults revealed an improvement of up to 15% [33,34,35]. Thus, our results are promising regarding considerable behavioral change at an early stage. Teacher-based stand-up prompting with a minimal target can be seen as beneficial means to motivate children to increase their standing time. In this regard, DeRuiter et al. [36] emphasized that a behavior change could be transferred into different areas of life and that an interrelationship of multiple behavioral changes exists. Our results of increased standing time in lessons do not allow a transfer to standing times during class breaks, because the percentage of standing time in the CG was comparable with IG times. Probably, the intervention period was not sufficient.

Additionally, we found that pupils were able to undertake similar standing time recordings compared to the external observation. The high convergence between the self-report and external report was supported by applying a control sheet for self-report data, which enables real-time recording. Jeffrey et al. [37] examined whether real-time self-reported adherence is an accurate measurement of device usage during a clinical trial by comparing it to electronic recordings of a vibrating platform for the treatment of osteoporosis and showed a high agreement between the two measures (ICC = 0.96). Real-time assessment is simple, inexpensive, time saving and less error prone. This type of assessment is preferred in comparison to retrospective measurement instruments, because it is much more accurate. On the other hand, it also requires some discipline to constantly fill out a self-report sheet, which is probably not appropriate for longer periods of time. Furthermore, it needs to be mentioned that the most accurate way to measure physical activity is to use objective measurements such as ActiGraph [38].

In addition to the problem of recall bias in retrospective studies of physical activity [39], retrospective measurements have the problem of social desirability, which means that in the context of physical activity, activity levels are overestimated and sedentary activity is underestimated [40,41]. However, the issue of social desirability must also be considered in the present study, since the documentation by external observers raises the pressure to document correctly. Nevertheless, self-reporting is a suitable, simple and easily implementable method for future large randomized controlled trials (RCTs) no complicated measures with external persons or accelerometers are needed when participants record reliably by themselves.

For changes in cognitive abilities in the context of the use of standing desks in children there is one comparable study using a similar approach to date [42]. They found that continued utilization of standing desks for 27 weeks was associated with significant improvements in executive function and working memory in 14-year-olds. Different to our study, they had no comparison group to evaluate the benefits of the standing desk intervention against a control. In our study the assumed improvement of cognitive performance due to a higher physical activity level could be confirmed for the digit span task only descriptively. These improvements for the IG compared to the CG show higher effect sizes. Due to the small sample size and the short intervention period this result can be seen as an interesting preliminary result.

In larger and longer future studies it might be worth looking at the effects of longer standing times on cognitive parameters in more detail. Our data suggest that potential effects may be more likely to be found in the short-term working memory than the inhibitory control. It would be interesting to investigate specific reasons for this assumption.

Our findings are in line with the results of the meta-analysis of Torbeyns et al. [21], who found that the effects of active workstations are a decrease in sitting time and no detrimental effect on work performance, cognitive function and computer task performance.

It can be summarized that a daily use of 41 min of the standing desk during 11 weeks, including 13 min longer standing time for the IG in comparison to the CG, is not sufficient to affect the cognitive performance in children considerably. On the other hand, no negative effects could be found. This result can be underlined by Koepp et al. [43], who showed no negative effects on concentration and possible inconveniences associated with standing desks in the classroom.

The study results comprise several limitations that need to be considered. As the study was designed as a pilot project, only two classes from two schools were included. Thus, a comparatively small sample size resulted. Nevertheless, the found effects were medium to large. Another sample with a larger variety of age groups, i.e., spread over a larger geographical region, is needed to confirm the results and would be interesting, because students aged 10 to 12 years are still very much active in comparison to students of higher classes. Ridgers et al. [44] showed that during breaks (recess and lunchtime) older adolescents engaged in less moderate-to-vigorous physical activity than younger adolescents. Therefore, it should be tested whether a standing desk intervention among older students may achieve greater effects.

The group assignment (IG vs. CG) of the two classes was non-randomized and the motivation could vary between intervention and control teachers. Nevertheless, the rural setting, the educational concept, the school grounds and sample characteristics were comparable.

Our data did not allow a comparison with the objectively measured data by ActiGraph since the data relate to different measurement periods. Previous studies summarized in a systematic review attest an overestimation of subjectively measured PA and an underestimation of sedentary behavior in comparison to objectively measured data [38,45]. Furthermore, in other contexts there are studies summarizing low validity for self-reported data and poor correlations with objective methods, for example self-reported data concerning the body mass index [46]. As the concordance between the self-report and external observation was high in our study, we hypothesize that the validity between real-time assessment and objective measurement in our sample was high, because a high correlation between objective measurement and external assessment is proved by several studies [47,48]. In order to draw a final conclusion about the differences between the several measurement methods, a study comparing all methods (retrospective questionnaires, real-time self-reports, external observations and objective measurement) is needed.

The fact that the teacher reminded the students of the minimum target of standing time can be viewed critically in the context of self-motivation and self-efficacy. On the one hand, it may be helpful to use cues when self-motivation is not sufficient and a new behavior needs to be learned. In addition, this enforces physical education and leads to a higher health awareness. Therefore, it is essential to establish standing settings at an early stage. On the other hand, a change in behavior will only be maintained in the long run, if the person is intrinsically motivated [49]. Future studies should examine how much the standing time increases when there are no further external specifications. It should be noted that even small changes in short survey periods can result in meaningful positive long-term effects. An increase of physical activity needs to be enforced not only in school settings, but also in leisure time. Additionally, other variables could be interesting moderators of our findings, such as teacher commitment and motivation, school concept and gender. Identifying further variables is important to enhance the development of tailored interventions to reduce sitting time and improve cognitive performance.

## 5. Conclusions

Our finding of a high level of sitting time in children in class emphasizes the need for further action to increase physical activity levels and to improve cognitive performance. With this study, we were able to show that the implementation of standing desks is worthwhile because it reduces the sitting time or the standing time may be increased, and the cognitive performance did not decline, rather slightly improved. In addition both teacher and pupils described qualitatively an improvement of their concentration, even though the implementation of standing desks was challenging. Nonetheless, schools need different interventions that are tailored to increase the physical activity of children and adolescents. There are different ways to reorganize and rebuild the school setting to provide a more “active environment” in the sense of structural prevention approaches [50].

For school health in general we argue from our study that the school setting provides high potential to decrease sitting by increasing standing time. In this context, standing desks are one opportunity to increase physical activity and academic performance, but also involve high financial and organizational challenges, even though appropriate conditions exist that can lead to a successful implementation of standing desks, like special class rooms equipped with standing desks or height adjustable desks to change position. Furthermore, children have to be reminded frequently to use these desks. We would recommend using a direct feedback method for the purpose of self-monitoring to initiate readjustments of physical activity and higher self-motivation. The named aspects could improve health consciousness and associated outcomes in the long run. The earlier health consciousness is developed concerning physical activity in daily life, the better. School represents an ideal platform to establish healthy life habits and the school setting makes it possible to reach a large range of children easily.

In conclusion, standing desks should be considered as a valuable contribution to programs engaged in prevention and health promotion to reduce sitting times and the risk of a number of chronic diseases as well as to improve cognitive performance, but they cannot be regarded as the only method.

## Figures and Tables

**Table 1:** Sample characteristics.

Mean Scores (SD)	IG (*n* = 19)	CG (*n* = 19)
age, years	10.8 (0.6)	10.8 (0.8)
weight, kg	39.3 (9.1)	40.8 (12.1)
height, cm	147.1 (7.8)	147.2 (8.2)
body mass index, kg/m^2^	18.0 (2.8)	18.6 (4.3)
sex, *n* (%)		
male	9 (47.4)	13 (68.4)
female	10 (52.6)	6 (31.6)

**Table 2:** Statistical values for daily sitting time, standing, and walking time during lessons and breaks measured by ActiGraph in IG (*n* = 19) and CG (*n* = 19).

Physical Activity	Group	Mean Time in % (SD)	Mean Time in Min (SD)	Mean Difference (95% CI)	*t*-Value (df)	*p*-Value	Effect Size d
Sitting time lesson	IG	68.2 (5.7)	172.1 (19.7)	12.7 (1.6; 23.9)	2.32 (36)	0.03 *	0.75
	CG	73.7 (5.4)	184.9 (13.7)				
Standing time lesson	IG	23.9 (5.6)	60.5 (15.1)	−13.4 (−22.3; −4.5)	−3.06 (36)	0.004 **	0.99
	CG	18.8 (4.7)	47.1 (11.6)				
Walking time lesson	IG	7.9 (2.5)	19.9 (6.3)	−1.0 (−4.6; 2.6)	−0.57 (36)	0.57	0.18
	CG	7.5 (1.7)	18.9 (4.4)				
Sitting time break	IG	32.3 (10.7)	7.5 (2.5)	−1.0 (−2.4; 0.4)	−1.48 (36)	0.15	0.47
	CG	34.3 (8.2)	6.5 (1.6)				
Standing time break	IG	37.3 (12.2)	8.8 (3.1)	−2.5 (−4.0; −0.9)	−3.15 (36)	0.003 **	1.04
	CG	33.4 (7.6)	6.3 (1.4)				
Walking time break	IG	30.4 (13.8)	7.0 (3.0)	−0.9 (−2.7; 0.8)	−1.05 (36)	0.30	0.34
	CG	32.3 (11.7)	6.1 (2.2)				

* *p* ≤ 0.05; ** *p* ≤ 0.01; SD = standard deviation, CI = Confidence interval, df = degrees of freedom.

**Table 3:** Main results for the digit span task and Eriksen flanker task: Statistical values for IG and CG (*n* = 38).

Cognitive Function	Group	Mean (SD) Pre	Mean (SD) Post	Mean Difference (95% CI)	Effect Size d	*p*-Value Time	*p*-Value Group	*p*-Value Time × Group
Forward digit span (Mean)								
Number of correct trials	IG	4.8 (1.5)	5.5 (1.4)	−0.7 (−1.6; 0.3)	0.48	0.07	0.59	0.50
	CG	4.8 (1.3)	5.2 (1.7)	−0.3 (−1.0; 0.3)	0.26			
Maximum sequence length	IG	4.8 (0.8)	5.2 (0.7)	−0.4 (−0.9; 0.1)	0.48	0.29	0.58	0.16
	CG	5.0 (0.7)	4.9 (0.7)	0.1 (−0.3; 0.4)	0.09			
Backward digit span (Mean)								
Number of correct trials	IG	5.0 (1.2)	5.7 (1.8)	−0.7 (−1.4; 0.0)	0.46	0.07	0.56	0.74
	CG	4.8 (1.7)	5.3 (1.9)	−0.5 (−1.6; 0.6)	0.26			
Maximum sequence length	IG	4.1 (0.8)	4.4 (1.0)	−0.4 (−0.8; 0.1)	0.41	0.16	0.28	0.57
	CG	3.9 (0.9)	4.1 (1.0)	−0.2 (−0.8; 0.5)	0.17			
Eriksen flanker reaction time (ms)								
Congruous	IG	476 (99)	451 (119)	25 (−18; 69)	0.23	0.04 *	0.92	0.95
	CG	474 (89)	447 (82)	27 (−3; 57)	0.32			
Incongruous	IG	546 (127)	518 (120)	28 (−27; 83)	0.23	0.07	0.85	0.95
	CG	541 (99)	510 (88)	30 (−7; 67)	0.32			
Eriksen flanker accuracy (%, range 0–1)								
Congruous	IG	0.87 (0.17)	0.92 (0.16)	−0.05 (−0.10; 0.01)	0.29	0.01 *	0.52	0.93
	CG	0.90 (0.14)	0.95 (0.06)	−0.05 (−0.09; 0.00)	0.42			
Incongruous	IG	0.74 (0.15)	0.79 (0.17)	−0.05 (−0.11; 0.00)	0.32	0.06	0.82	0.75
	CG	0.76 (0.19)	0.80 (0.15)	−0.04 (−0.12; 0.04)	0.22			

* *p* ≤ 0.05; IG = intervention group, CG = control group, SD = standard deviation, CI = Confidence interval, df = degrees of freedom.
